# Robot‐Assisted Salvage Prostatectomy: External Validation of the EAU Selection Criteria and Identification of the Optimal Candidate: A Junior ERUS/YAU Collaborative Study

**DOI:** 10.1002/pros.70048

**Published:** 2025-09-24

**Authors:** Mike Wenzel, Christoph Würnschimmel, Arjun Nathan, Marcio Covas Moschovas, Christian Wagner, Giorgio Calleris, Fabrizio Di Maida, Juan Gomez Rivas, Carlo Andrea Bravi, Ruben De Groote, Federico Piramide, Filippo Turri, Keith Kowalczyk, Gopal Sharma, Iulia Andras, Edward Lambert, Nikolaos Liakos, Danny Darlington, Marco Paciotti, Gabriele Sorce, Philipp Mandel, Antonio Galfano, Senthil Nathan, Giancarlo Marra, Paolo Dell'Oglio, Alexandre Mottrie, Felix K. H. Chun, Vipul Patel, Alberto Breda, Alessandro Larcher, Junior ERUS/EAU‐YAU Working Group on Robot‐Assisted Surgery

**Affiliations:** ^1^ Department of Urology Goethe University Hospital Frankfurt Frankfurt am Main Germany; ^2^ Department of Urology Luzerner Kantonsspital Lucerne Switzerland; ^3^ University College London Hospitals NHS Foundation Trust London UK; ^4^ AdventHealth Global Robotics Institute Celebration Florida USA; ^5^ Department of Urology, Pediatric Urology and Uro Oncology Prostate Center Northwest, St. Antonius‐Hospital Gronau Germany; ^6^ Department of Surgical Sciences Urology Clinic, University of Turin and Città della Salute e della Scienza Turin Italy; ^7^ Department of Experimental and Clinical Medicine Unit of Oncologic Minimally‐Invasive Urology and Andrology, Careggi Hospital University of Florence Florence Italy; ^8^ Department of Urology Hospital Clínico San Carlos Madrid Spain; ^9^ Department of Urology The Royal Marsden NHS Foundation Trust London UK; ^10^ Department of Urology Onze‐Lieve‐Vrouwziekenhuis Hospital Aalst Belgium; ^11^ ORSI Academy Ghent Belgium; ^12^ Department of Oncology Division of Urology, San Luigi Gonzaga Hospital University of Turin Turin Italy; ^13^ Department of Urology ASST Santi Paolo e Carlo University of Milan Milan Italy; ^14^ Department of Urology MedStar Georgetown University Hospital Washington DC USA; ^15^ Department of Urologic Oncology Medanta – The Medicity Gurgaon India; ^16^ Department of Urology Iuliu Hatieganu University of Medicine and Pharmacy Cluj‐Napoca Romania; ^17^ Department of Urology Ghent University Hospital Ghent Belgium; ^18^ Department of Urology Medical Centre of the University of Freiburg, Faculty of Medicine Freiburg Germany; ^19^ Department of Urology Stokes Centre for Urology, Royal Surrey County Hospital Guildford UK; ^20^ Department of Urology Humanitas Research Hospital – IRCCS Rozzano Italy; ^21^ Department of Urology IRCCS San Raffaele Hospital Milan Italy; ^22^ Urology Department ASST Grande Ospedale Metropolitano Niguarda Milan Italy; ^23^ Department of Urology, Universitat Autonoma de Barcelona Fundació Puigvert Barcelona Spain

**Keywords:** BCR, brachytherapy, MFS, recurrent prostate cancer, SRARP

## Abstract

**Background:**

EAU guidelines recommend salvage radical prostatectomy (sRP) only in highly selected patients with recurrent prostate cancer in experienced centers.

**Methods:**

The Junior ERUS/Young Academic Urologist Working Group on Robot‐Assisted Surgery conducted a multicentric project to investigate biochemical recurrence‐free (BCR), metastases‐free (MFS), and overall survival (OS) outcomes in robotic sRP patients stratified according to EAU criteria.

**Results:**

Of 180 patients, 49% fulfilled EAU criteria. Patients not fulfilling EAU criteria more frequently underwent focal therapy as primary treatment (53% vs. 33%) and exhibited significantly higher rates of pT3–4 (70% vs. 48%), positive surgical margins (48% vs. 24%), and pathological Gleason score 8–10 (72% vs. 48%, all *p* < 0.01), with no differences in postoperative complications. Rates of PSA persistence were significantly higher in patients not fulfilling EAU criteria (16% vs. 0%, *p* < 0.001). Regarding BCR, patients not fulfilling EAU criteria harbored significantly worse BCR‐free survival (hazard ratio (HR): 1.96, *p* = 0.046) with 24‐ and 48‐month BCR‐free survival rates of 81.7% and 73.9% vs. 65.0% and 58.5% for patients fulfilling EAU criteria. After multivariable adjustment, patients not fulfilling EAU criteria harbored higher risk of BCR (HR: 2.94, *p* = 0.045). Regarding MFS and OS outcomes, no significant differences were observed in the comparison between both groups. Incorporating presalvage surgery features into a new classification yielded better discrimination for BCR analysis, but were comparable to EAU criteria for MFS and OS outcomes.

**Conclusions:**

The majority of patients do not fulfill EAU criteria, and even more so after focal therapy. These patients harbor worse BCR rates after robotic sRP. However, within our short‐term follow‐up, no differences in MFS and OS were observed.

## 1 Introduction

In recurrent prostate cancer after initial treatment with radiation therapy or focal therapy in curative intent, salvage radical prostatectomy (sRP) remains a potentially curative secondary treatment option [1, 2, 3, 4]. However, sRP is a rarely performed procedure with, for example, only 428 performed cases in the United States between 2004 and 2016 due to high perioperative morbidity and poor functional outcomes [5, 6, 7].

EAU guidelines recommend sRP only in selected patients, following thorough scrutiny based on the following criteria: life expectancy of at least 10 years, a presalvage PSA < 10 ng/mL, pathological ISUP Grade Group 2/3 at initial prostate cancer diagnosis with organ‐confined disease (cT1–2), and cN0 and cM0 stage prior to sRP [1]. These criteria rely on a meta‐analysis from 2012, including data on sRP series between 1988 and 2011 [8]. Due to the rarity of the procedure, literature validating these EAU criteria are scant. Currently, one single‐center report from 2016 included 55 patients treated between 2007 and 2012 and a more recent multicenter study relying on 1265 patients treated with sRP between 2000 and 2021 [9, 10] are available. Due to global adoption of robotic surgical systems, sRP is nowadays mostly performed robotically [11, 12]. However, data on exclusively robotically treated sRP are even more scant and often limited by sample size [13, 14, 15, 16].

We addressed this knowledge gap and conducted a multicenter study within high‐volume robotic prostate cancer centers across the world and within the Junior ERUS/Young Academic Urologist Working Group on Robotics in Urology. We hypothesized that significant differences in oncological outcomes such as biochemical recurrence (BCR)‐free survival, metastasis‐free survival (MFS), or overall survival (OS) rates exist following stratification according to EAU criteria for robotic salvage radical prostatectomy (s‐RARP) patients. Moreover, we aimed to investigate other perioperative predictors for a better patient stratification with the aim to define the optimal candidates for s‐RARP.

## 2 Materials and Methods

### 2.1 Study Population

With approval from the Local Ethics Committee at the Primary Investigator's Center (Approval Number: SUG‐5‐2018) and in compliance with the Declaration of Helsinki, a multicenter study was conducted by the Junior ERUS/Young Academic Urologist Working Group on Robotics in Urology on patients who underwent s‐RARP after initial treatment for prostate cancer at 13 centers across the world between 2007 and 2023. For analyses, the study relied on the available multicenter database of s‐RARP patients, as recently reported (*n* = 445) [17, 18]. Patients with metastatic disease and castration‐resistant prostate cancer were excluded. Moreover, patients were excluded if missing necessary data for stratification according to EAU criteria for salvage RARP. This study inclusion criteria yielded 180 patients who received s‐RARP (range *n* = 1–44 patients/center).

### 2.2 Definition of EAU Criteria

Patients were classified in two groups if they fulfilled versus did not fulfill the EAU criteria for salvage RARP. EAU criteria were a presalvage PSA < 10 ng/mL, pathological ISUP Grade Group 2/3 at initial prostate cancer diagnosis with organ‐confined disease (cT1‐2), staging prior to sRP was cN0/cNx and cM0, in accordance with EAU guidelines [1].

### 2.3 Data Collection and Oncological Outcomes

All retrospectively collected data such as baseline patient and tumor, as well as surgical characteristics or oncological outcomes of s‐RARP patients, were anonymously sampled. Positive surgical margin (PSM) rates and PSA persistence were collected from patient files. PSA persistence was defined as a post s‐RARP PSA level above ≥ 0.1 ng/mL at first measurement after surgery [19]. BCR was defined as a PSA rise above ≥ 0.2 ng/mL after s‐RARP in accordance with EAU criteria [1]. Patients with PSA persistence after s‐RARP were excluded from BCR analyses. MFS was defined as the time from s‐RARP to the occurrence of the first nonregional metastasis. OS was defined as the time from surgery to death from any cause. All staging modalities were performed either with conventional or molecular PSMA‐PET/CT imaging.

### 2.4 Statistical Analysis

Descriptive statistics included frequencies and proportions for categorical used variables. Medians and interquartile ranges (IQR) were reported for all applied continuously coded variables. The *χ* ^2^ test was used to test for statistical significance in proportions' differences. In addition, the *t*‐test and Kruskal–Wallis test examined the statistical significance of distributions' differences. Postoperative complications were sampled and stratified according to Clavien–Dindo.

For oncological BCR, MFS, and OS outcome analyses, Kaplan–Meier curves analyses depicted s‐RARP patients stratified according fulfilling versus nonfulfilling EAU criteria. Moreover, univariable, as well as multivariable Cox regression models were applied to adjust for baseline and pathological tumor characteristics such as age at surgery, time interval between initial primary prostate cancer treatment and surgery for relapsing prostate cancer, PSMs, primary treatment (focal therapy vs. radiation therapy), pathological stage after surgery, and pathological Gleason score. For OS analyses, due to a few events and to prevent overfitting the model, no further adjustment for age at surgery and time interval between initial primary prostate cancer treatment and surgery could be made. Moreover, we additionally elaborated the patient/tumor characteristics, which violated EAU criteria and stratified patients accordingly into not‐fulfilling EAU criteria due to Gleason score versus other reasons, separate cancer‐control measurements as described above were performed for these patients.

In the last step of the analyses, we aimed to improve EAU criteria by incorporating and using other predictive variables in uni‐ and multivariable analyses for oncological outcomes analyses. For comparisons to EAU criteria, c‐index calculations were applied. All tests were two‐sided with a level of significance set at *p* < 0.05. R software environment for statistical computing and graphics (Version 3.4.3) was used for all analyses.

## 3 Results

### 3.1 Baseline Characteristics of s‐RARP Patients

Overall, 180 patients qualified for inclusion of the current study with a median follow‐up of 21 months (IQR: 9–39 months). Median time between initial prostate cancer treatment and s‐RARP was 49 months (Table 1) and patients' median age was 69 years (IQR: 65–73) prior to salvage surgery with a median PSA level of 7.7 ng/mL (IQR: 3.1–12.7 ng/mL).

**Table 1:** Characteristics of 180 robotic salvage radical prostatectomy (sRP) patients stratified according to fulfilling EAU criteria.

Characteristic	*N*	Overall, *N* = 180^a^	EAU criteria fulfilled, *N* = 89 (49%)^a^	EAU criteria not fulfilled, *N* = 91 (51%)^a^	*p* b
Month between initial PCa and sRP	151	49 (20, 87)	59 (24, 94)	43 (19, 72)	0.088
Age initial PCa	108	63 (59, 68)	63 (59, 68)	64 (60, 68)	0.5
Age sRP	148	69 (65, 73)	70 (66, 73)	69 (64, 72)	0.4
BMI	149	27.4 (25.1, 30.0)	27.1 (25.1, 29.0)	27.8 (25.2, 31.7)	0.13
CCI initial PCa	81	1 (0, 4)	4 (4, 5)	0 (0, 1)	< 0.001
CCI sRP	88	4 (3, 5)	4 (3, 5)	4 (2, 5)	0.11
PSA initial PCa	94	7 (6, 10)	7 (6, 9)	8 (6, 15)	0.2
PSA prior sRP	155	7.7 (3.1, 12.7)	3.5 (2.1, 5.9)	12.0 (10.3, 16.5)	< 0.001
Positive cores initial PCa	68	4 (2, 5)	4 (2, 5)	5 (2, 6)	0.2
Positive cores prior sRP	74	4.0 (2.0, 7.8)	5.0 (2.0, 8.0)	4.0 (2.0, 7.0)	0.9
Tumor infiltration initial PCa, %	37	50 (20, 80)	50 (20, 80)	45 (29, 70)	> 0.9
Tumor infiltration prior sRP, %	58	50 (19, 80)	50 (20, 80)	45 (19, 80)	0.7
ADT	155	108 (70%)	51 (75%)	57 (66%)	0.2
ADT duration, months	16	12 (6, 24)	12 (8, 12)	9 (5, 24)	0.7
LND performed	180	129 (72%)	74 (83%)	55 (60%)	< 0.001
LND: number of nodes	131	10 (5, 16)	12 (6, 16)	8 (4, 13)	0.3
EBL	145	200 (100, 300)	200 (100, 300)	200 (100, 300)	0.13
OR time	175	164 (130, 188)	160 (130, 194)	170 (134, 180)	> 0.9
Catheter days	118	10 (5, 19)	10 (5, 14)	14 (10, 33)	0.001
ECOG 1–2	49	21 (43%)	12 (41%)	9 (45%)	0.8
Initial cT ≥ 3–4	105	3 (2.9%)	0 (0%)	3 (10%)	0.022
cT prior sRP ≥ 3–4	132	34 (26%)	6 (12%)	28 (34%)	< 0.001
Gleason score initial PCa	117				< 0.001
6		54 (46%)	48 (57%)	6 (18%)	
7		46 (39%)	36 (43%)	10 (30%)	
8–10		17 (15%)	0 (0%)	17 (52%)	
Gleason score prior sRP	160				< 0.001
6		25 (16%)	19 (26%)	6 (7.0%)	
7		90 (56%)	44 (59%)	46 (53%)	
8–10		45 (28%)	11 (15%)	34 (40%)	
Primary therapy	177				0.007
FT		77 (44%)	29 (33%)	48 (53%)	
RT		100 (56%)	58 (67%)	42 (47%)	
Nerve sparing	179	93 (52%)	49 (55%)	44 (49%)	0.4
pT3–4	178	105 (59%)	42 (48%)	63 (70%)	0.003
pN1	162	30 (19%)	15 (20%)	15 (17%)	0.7
Pathological Gleason score	113				0.007
6		3 (2.7%)	1 (1.6%)	2 (4.0%)	
7		44 (39%)	32 (51%)	12 (24%)	
8–10		66 (58%)	30 (48%)	36 (72%)	
Complications CD	30				0.2
1		18 (60%)	5 (42%)	13 (72%)	
2		4 (13%)	2 (17%)	2 (11%)	
3		5 (17%)	3 (25%)	2 (11%)	
3a		1 (3.3%)	0 (0%)	1 (5.6%)	
3b		2 (6.7%)	2 (17%)	0 (0%)	

Abbreviations: ADT, androgen deprivation therapy; BMI, body mass index; CCI, Charlson Comorbidity Index; CD, Clavien–Dindo; ECOG, Eastern Cooperative Oncology Group; EBL, estimated blood loss; GS, Gleason score; LND, lymph node dissection; OR, operating room; PCa, prostate cancer; · PSM, positive surgical margin; PSA, prostate‐specific antigen.

^a^ Median (IQR); *n* (%).

^b^ Kruskal–Wallis rank sum test; Fisher's exact test; Pearson's *χ* ^2^ test.

### 3.2 EAU Criteria in s‐RARP Patients

After stratifying s‐RARP patients according to EAU criteria, 49% fulfilled EAU criteria (Table 1). Of patients not fulfilling EAU criteria, 19% were due to initial Gleason score 8–10 (+/−) additional reason versus 81% due to other non‐Gleason score reasons (cT stage, PSA, cN stage).

In comparison between patients fulfilling versus not fulfilling EAU criteria, patients not fulfilling EAU criteria harbored higher median PSA prior to salvage surgery (12.0 vs. 3.5 ng/mL, *p* < 0.001). Conversely, baseline characteristics such as age at primary prostate cancer diagnosis, age at salvage treatment, and month between primary treatment and salvage treatment were comparable (all *p* ≥ 0.09). However, Gleason score at initial cancer diagnosis and prior to s‐RARP significantly differed regarding proportions of Gleason score 8–10 (0% and 15% for EAU criteria fulfilled vs. 52% and 40% for EAU criteria not fulfilled, both *p* < 0.001). Regarding primary treatment for prostate cancer, rate of focal therapy was significantly higher in patients not fulfilling EAU criteria (53% vs. 33%, *p* < 0.01).

Regarding surgical characteristics, patients fulfilling EAU criteria significantly more frequently underwent lymph node dissection (83% vs. 60%, *p* < 0.001). Moreover, rates of pT3–4 stage (70% vs. 48%), and pathological Gleason score 8–10 (72% vs. 48%) were significantly higher in patients not fulfilling EAU criteria (all *p* < 0.01).

Regarding postoperative characteristics, no differences in complications were observed and the majority of complications were limited to Clavien–Dindo Grade I–II. However, rates of PSA persistence were significantly higher in patients not fulfilling EAU criteria (16% vs. 0%, *p* < 0.001).

### 3.3 Oncological Outcomes Regarding EAU Criteria

PSM rates were significantly higher in patients not fulfilling EAU criteria (48% vs. 24%), similar to PSA persistency (16% vs. 0%, both *p* < 0.001, Table 2). BCR rates were also higher for patients not fulfilling EAU criteria (30 vs. 15%), without reaching significance (*p* = 0.07).

**Table 2:** Oncological outcomes of robotic salvage radical prostatectomy (sRP) patients stratified according fulfilling EAU criteria and reason for not fulfilling (NF) EAU criteria.

Characteristic	*N*	Overall rate^a^	EAU criteria fulfilled^a^	EAU criteria not fulfilled^a^	*p* b	*N*	NF: Gleason score^a^	NF: other reason^a^	*p* b
PSM	178	64 (36%)	21 (24%)	43 (48%)	< 0.001	89	8 (47%)	35 (49%)	1
PSA persistence	177	14 (7.9%)	0 (0%)	14 (16%)	< 0.001	90	1 (6.3%)	13 (20.3%)	0.5
BCR	147	38 (22%)	14 (19%)	23 (31%)		74	7 (50%)	16 (27%)	
Metastatic disease	113	11 (9.7%)	6 (13%)	5 (7.7%)		65	3 (42%)	2 (3.4%)	
Overall mortality	174	14 (8.0%)	6 (7.8%)	8 (9.1%)		88	2 (13%)	6 (0.3%)	

Abbreviations: BCR, biochemical recurrence; N, representing the number of patients with available information regarding the specific oncologic outcome; PSA, prostate‐specific antigen; PSM, positive surgical margin.

^a^ *n* (%).

^b^ Kruskal–Wallis rank sum test; Fisher's exact test; Pearson's *χ* ^2^ test.

**Figure 1:**
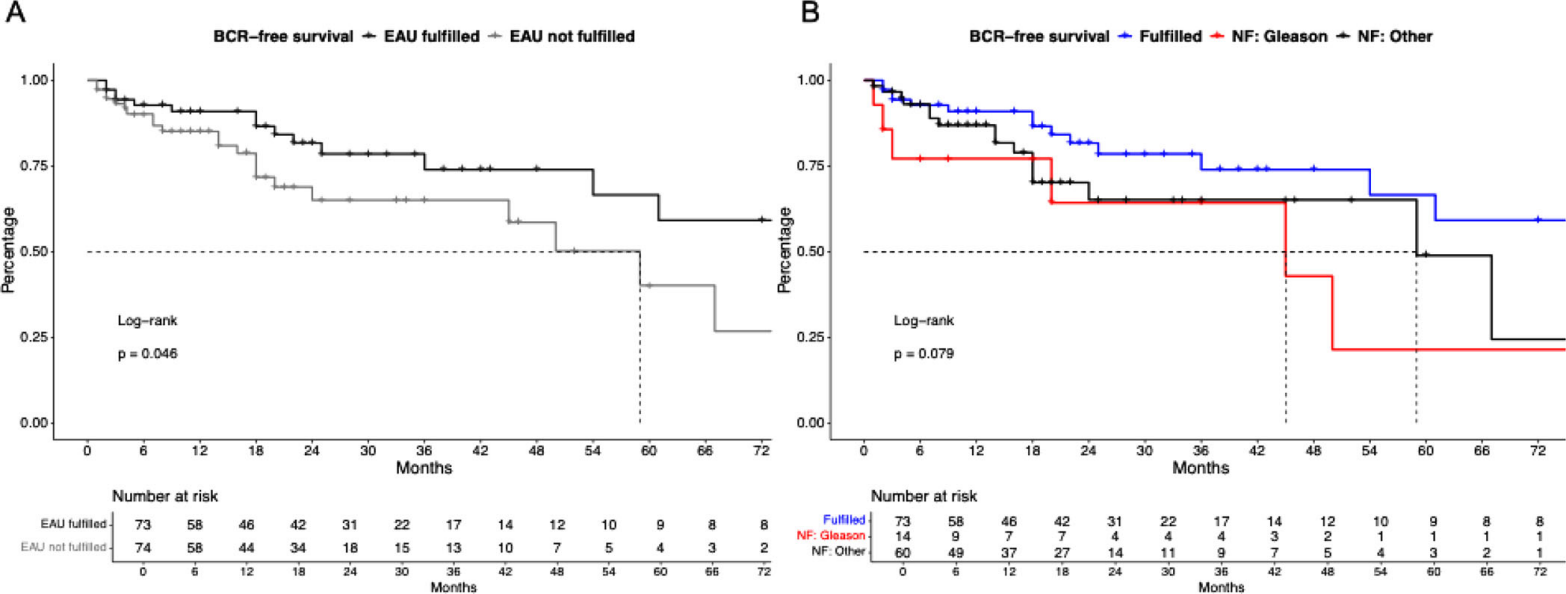
Kaplan–Meier curve depicting biochemical recurrence (BCR)‐free survival in robotic salvage radical prostatectomy patients stratified according to fulfilling EAU criteria (A) and the reason for not fulfilling (NF) EAU criteria (B). [Color figure can be viewed at wileyonlinelibrary.com]

In Kaplan–Meier BCR analyses after stratification according EAU criteria, patients not fulfilling EAU criteria harbored significantly worse BCR‐free survival (Figure 1A), with a hazard ratio (HR) of 1.96 (*p* = 0.046) and 24‐ and 48‐month BCR‐free survival rates of 81.7% and 73.9% versus 65.0% and 58.5% for patients fulfilling versus not fulfilling EAU criteria. Median BCR‐free survival was not reached (NR) versus 59 months for fulfilling versus not fulfilling EAU criteria. After multivariable adjustment, patients not fulfilling EAU criteria were independently associated with a higher risk of BCR (HR: 2.94, *p* = 0.045, Supporting Information S1: Table 1).

In BCR comparison of patients not fulfilling EAU criteria (Figure 1B), median BCR‐free survival was 45 versus 59 months for criteria of Gleason score versus other reasons. Gleason score was in univariable Cox models significantly associated with higher BCR risk, relative to patients fulfilling EAU criteria (HR: 2.71, *p* = 0.041), while patients with other reasons were not. No differences in multivariable models were recorded.

**Figure 2:**
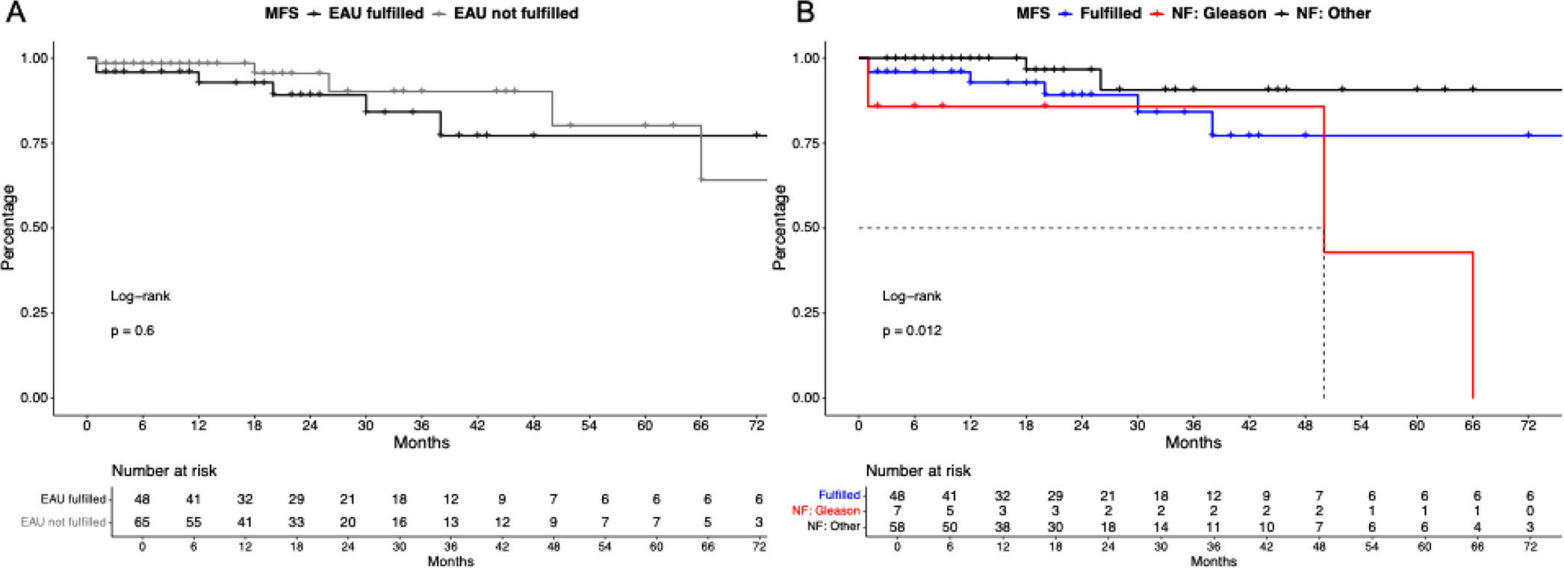
Kaplan–Meier curve depicting metastasis‐free survival (MFS) in robotic salvage radical prostatectomy patients stratified according to fulfilling EAU criteria (A) and the reason for not fulfilling (NF) EAU criteria (B). [Color figure can be viewed at wileyonlinelibrary.com]

Regarding MFS outcomes, no significant differences were observed in the comparison between patients fulfilling versus not fulfilling EAU criteria (Figure 2A, *p* = 0.6) with 24 and 48 MFS rates of 89.1% and 77.2% versus 95.5% and 90.2%. After multivariable adjustment, not fulfilling EAU criteria was not independently associated with a higher risk of metastasis (HR: 3.7, *p* = 0.2).

In comparison between patients not fulfilling EAU criteria (Figure 2B), median MFS was 50 months versus not reached for Gleason score versus other reasons. No significant differences in uni‐ or multivariable models were recorded.

**Figure 3:**
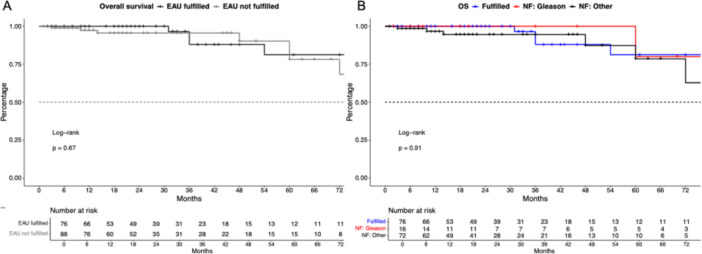
Kaplan–Meier curve depicting overall survival in robotic salvage radical prostatectomy patients stratified according to fulfilling EAU criteria (A) and the reason for not fulfilling (NF) EAU criteria (B). [Color figure can be viewed at wileyonlinelibrary.com]

Regarding OS outcomes, also no significant differences were observed in the comparison between patients fulfilling versus not fulfilling EAU criteria (Figure 3A, *p* = 0.67) with 24 and 48 OS rates of 100% and 88.0% versus 95.5% and 90.2%. After multivariable adjustment, not fulfilling EAU criteria was not independently associated with a higher risk of death (HR: 2.5, *p* = 0.3). In comparison between reason for not fulfilling EAU criteria, no differences were observed (Figure 3B).

### 3.4 Oncological Outcomes in New Classification

Instead of using only tumor characteristics from initial prostate cancer diagnosis, we created a model incorporating Gleason score prior to sRP (Gleason score ≤ 7 vs. 8–10) and time interval between primary prostate cancer treatment (< 12 vs. ≥ 12 months) as the strongest predictors from Cox regression models additionally to EAU criteria (Supporting Information S2: Table 2).

**Figure 4:**
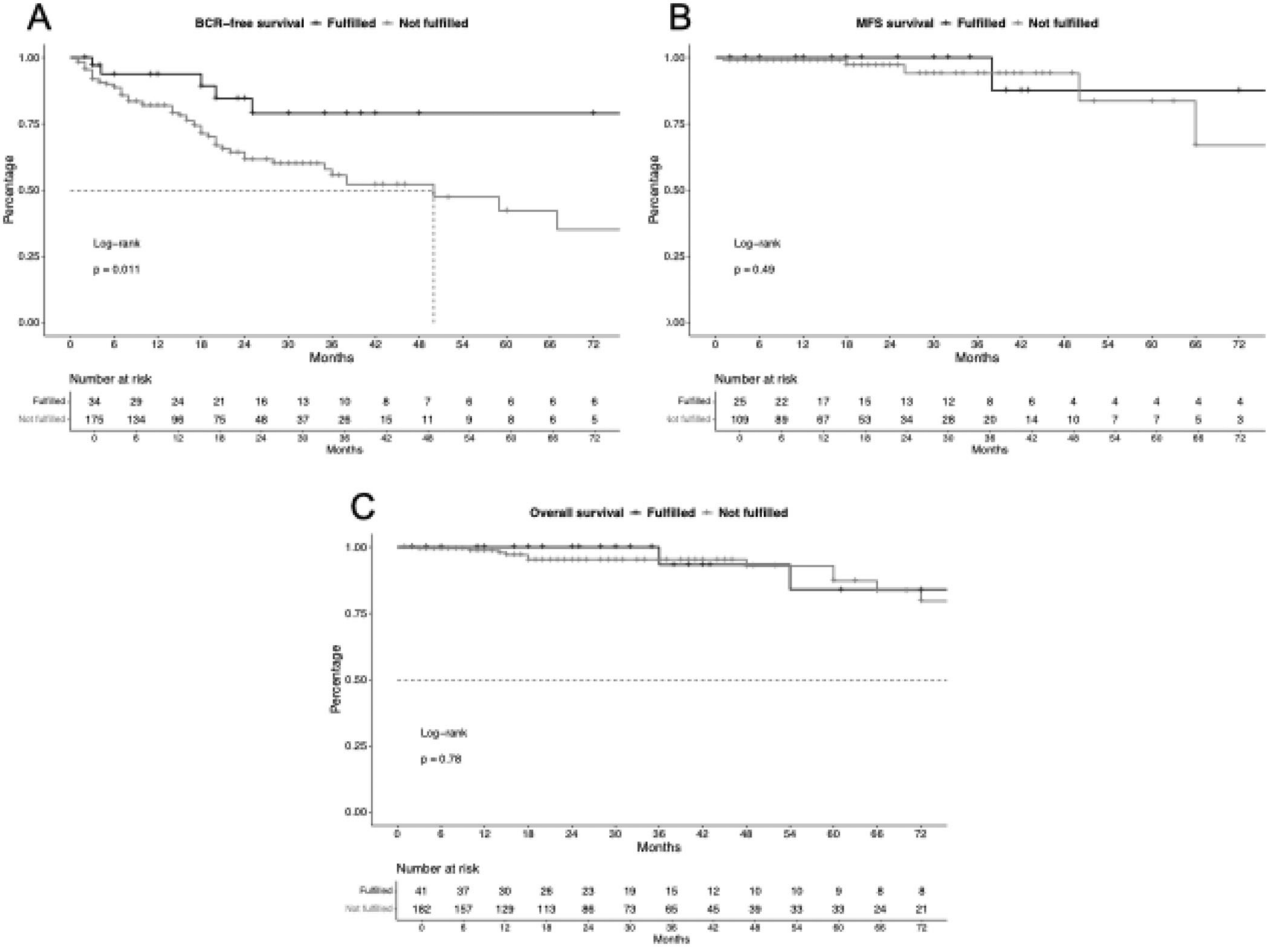
Kaplan–Meier curves depicting biochemical recurrence (BCR, A), metastasis‐free survival (MFS, B), and overall survival (C) in robotic salvage radical prostatectomy patients stratified to newly applied criteria.

Applying these criteria yielded better BCR‐free survival discriminations (Figure 4A) with a HR of 3.28 for not fulfilling these criteria (*p* < 0.01). Moreover, in multivariable Cox regression models adjusting for pathological characteristics (pT, Gleason score), PSM, and initial therapy, this model was an independent predictor of BCR (HR: 3.44, *p* = 0.02). In c‐index analyses, these criteria yielded a c‐index of 0.59 compared to EAU criteria with 0.57.

In MFS and OS analyses (Figure 4A,B), also no differences between fulfilling versus not fulfilling these criteria were observed with similar c‐indices as EAU criteria.

## 4 Discussion

We conducted a multicenter study within high‐volume robotic prostate cancer centers across the world and within the Junior ERUS/Young Academic Urologist Working Group on Robotics in Urology to assess and validate oncological outcomes of BCR‐free survival, MFS and OS in contemporary treated s‐RARP patients stratified according to EAU criteria. We hypothesized that EAU criteria sufficiently discriminate s‐RARP patients in a homogenous robotic cohort. We made several important observations.

First, we observed that despite EAU guidelines recommend sRP patients only in selected patients according to initial prostate cancer characteristics (cT stage and Gleason score), as well as characteristics at prostate cancer relapse (PSA, life expectancy, cN, cM stage), half (51%) of the patients within our study did not fulfill these criteria. However, these data are not surprising, compared to rates from previous reports. For example, in the first validation of the EAU criteria in 2016 with 55 patients from a single center cohort, also 42% did not fulfill EAU criteria [9]. Moreover, in the most recent multicenter validation by Calleris and colleagues 79% of all included 1030 sRP patients did not fulfill EAU criteria [10]. These low rates of patients fulfilling EAU criteria emphasize the need for alternative salvage treatments when patients with unfavorable prostate cancer do relapse in clinical practice. Usually in a clinical scenario, most physicians aim to pay the price for possibly worse functional outcomes only when good oncological outcomes are expected in salvage settings. With the approval of enzalutamide for patients with nonmetastatic hormone‐sensitive prostate cancer patients after initial radiation therapy and PSA relapse another nonsurgical salvage treatment option is newly available, when other salvage treatments are not considered and may reduce the number of performed sRPs in the future [20]. Further epidemiological analyses are needed within the upcoming years to show if selection criteria will change in sRP patients and the proportion of patients fulfilling EAU criteria undergoing sRP will rise.

When comparing baseline and prostate cancer characteristics of s‐RARP patients, patients not fulfilling EAU criteria harbored significantly worse characteristics such as PSA prior to salvage surgery, higher proportion of cT3–4 stage, and higher proportion of Gleason score 8‐10 prior to salvage treatment. These findings are not surprising since EAU criteria select patients, for example, with cT1–2 stage and Gleason score 6–7 at initial prostate biopsy. However, patients not fulfilling these initial criteria at prostate cancer diagnosis are obviously also of higher risk to harbor worse characteristics in case of relapsing prostate cancer of their high‐risk prostate cancer. These observations are in an agreement with previously published literature. For example, in the report by Mandel and colleagues, patients not fulfilling EAU criteria also harbored worse tumor characteristics prior to sRP [9]. However, it is of note that the rates of patients who initially underwent focal therapy for prostate cancer treatment more frequently did not fulfill EAU criteria. These observations are worrisome since they indicate that poor selection criteria were applied for initial prostate cancer treatment with focal therapy in patients with unfavorable features and high risk of relapsing. In consequence, due to risk of treatment failure, risk of insufficient patient selection, and unavailable long‐term data, EAU guidelines currently recommend focal therapy with HIFU or cryotherapy only in patients participating in trials or prospective registries [1, 21, 22, 23, 24]. Nonetheless, previous publications also demonstrated that feasible outcomes in patients with primary focal therapy and secondary sRP can be observed, irrespectively of whole gland or partial gland ablation [25].

Second, when oncological outcomes were compared, patients not fulfilling EAU criteria harbored significantly worse BCR‐free survival. This disadvantage was also observable after adjusting in multivariable Cox regression models for potential confounding variables with a 2.9‐fold higher risk of BCR. However, in MFS and OS analyses, no differences were observed between both groups. These observations are consistent with previous publications. For example, Mandel and colleagues also reported significant worse BCR‐free survival rates for patients not fulfilling EAU criteria [9]. However, comparing absolute BCR rates, Mandel and colleagues reported a 60‐month BCR‐free survival of 11.6% in patients not fulfilling EAU criteria, while within our study, after 48 months, 58.5% did not harbor BCR. Explanations for our substantially higher rates in BCR‐free survival rates within our contemporary cohort may be the homogenous cohort of robotically performed salvage procedures only and their corresponding advantages such as better surgical visibility or less blood loss [26, 27]. However, in contrast to our report, Calleris and colleagues found significantly worse MFS and OS rates in patients not fulfilling EAU criteria. Our lack of significance may be partially explained by too short follow‐up (median 21 months) and corresponding low rates of events and sample size limitations.

Finally, when efforts were made to improve the EAU criteria by incorporating additional baseline and tumor characteristics prior to s‐RARP, we observed only a marginal gain in discrimination for BCR in c‐index analyses (0.59 vs. 0.57). After multivariable adjustment, this new model also independently predicted worse BCR; however, given the low number of events, these results should be interpreted with caution. Importantly, both models showed limited discriminatory power, which restricts their immediate clinical applicability. In further MFS and OS analyses, no additional improvement over the EAU criteria was observed. Longer follow‐up and larger data sets will be required to refine selection criteria, potentially by integrating other relevant variables such as details of primary prostate cancer treatment.

In addition to the limitations stated above, data should be interpreted and acknowledged in its retrospective design. Moreover, the advantages of multicenter studies may also harbor the disadvantage of differences in data sampling and lack of some variables of interest which may introduce a bias. Moreover, the attempt to provide outcomes within a homogenous cohort of robotically treated patients of selected high‐volume centers may also limit the follow‐up period and the sample size to achieve statistical significance or clinical meaningful differences. The limited follow‐up duration, as well as low event rates for some of the reported cancer‐control outcomes, should be acknowledged in the interpretation of the current study. Finally, no data on patient‐reported outcomes were available, which may differ according to initial treatment [28, 29].

## 5 Conclusion

Taken together, important differences in contemporary robotically treated sRP patients exist when stratifying according to EAU criteria. The majority of patients do not fulfill these criteria, and those patients mainly undergo focal therapy for initial prostate cancer treatment. The most frequent reason for not fulfilling EAU criteria consists of non‐Gleason score reasons (cT stage, PSA, cN stage). These patients experience lower BCR‐free survival compared to patients fulfilling EAU criteria.

## Ethics Statement

The current study was in accordance with the Declaration of Helsinki. Local Review of the Ethics Board was approved.

## Consent

The authors have nothing to report.

## Conflicts of Interest

The authors declare no conflicts of interest.

## Supporting information


**Supplemental Table 1:** Univariable und multivariable Cox regression models predicting biochemical recurrence (BCR, A), metastatic‐free survival (MFS, B) and overall survival (OS, C) for salvage robotic radical prostatectomy (s‐RARP) patients, stratified according to fulfilling EAU criteria.

**Supplemental Table 2:** Comparison between current EAU criteria for selecting patients for salvage radical prostatectomy vs. those proposed within the current manuscript.

## Data Availability

Data are available for bona fide researchers who request it from the authors.
