# Supplementary material for: Robot‐Assisted Salvage Prostatectomy: External Validation of the EAU Selection Criteria and Identification of the Optimal Candidate: A Junior ERUS/YAU Collaborative Study
Source: Prostate. 2025 Sep 24;86(1):3–11. doi: 10.1002/pros.70048 (PMC12667225; doi:10.1002/pros.70048)
Supplement: Supplementary file 1 — Supplemental Table 1: Univariable und multivariable Cox regression models predicting biochemical recurrence (BCR, A), metastatic‐free survival (MFS, B) and overall survival (OS, C) for salvage robotic radical prostatectomy (s‐RARP) patients, stratified according to fulfilling EAU criteria. [file PROS-86-3-s001.docx]

|  | Univariable | | | Multivariable | | |
| --- | --- | --- | --- | --- | --- | --- |
| 1. BCR | **HR** | **CI** | **p value** | **HR** | **CI** | **p value** |
| EAU criteria fulfilled | **Ref.** | **-** | **-** | **Ref.** | **-** | **-** |
| EAU criteria not fulfilled* | 1.96 | 1.01-3.85 | 0.046 | 2.94 | 1.02-8.43 | 0.045 |
| 1. MFS |  |  |  |  |  |  |
| EAU criteria fulfilled | **Ref.** | **-** | **-** | **Ref.** | **-** | **-** |
| EAU criteria not fulfilled* | 0.72 | 0.22-2.38 | 0.6 | 3.72 | 0.48-28.67 | 0.20 |
| 1. OS |  |  |  |  |  |  |
| EAU criteria fulfilled | **Ref.** | **-** | **-** | **Ref.** | **-** | **-** |
| EAU criteria not fulfilled^+^ | 1.26 | 0.44-3.65 | 0.66 | 2.48 | 0.51-11.97 | 0.3 |

Adjustment in multivariable Cox regression models was made for:

*Months between initial treatment and s-RARP, type of primary treatment for prostate cancer; positive surgical margin, age at s-RARP, pT stage, Gleason score

^+^ type of primary treatment for prostate cancer; positive surgical margin, pT stage, Gleason score
