# Supplementary material for: Robot‐Assisted Salvage Prostatectomy: External Validation of the EAU Selection Criteria and Identification of the Optimal Candidate: A Junior ERUS/YAU Collaborative Study
Source: Prostate. 2025 Sep 24;86(1):3–11. doi: 10.1002/pros.70048 (PMC12667225; doi:10.1002/pros.70048)
Supplement: Supplementary file 2 — Supplemental Table 2: Comparison between current EAU criteria for selecting patients for salvage radical prostatectomy vs. those proposed within the current manuscript. [file PROS-86-3-s002.docx]

| ****EAU criteria**** | ****Newly proposed criteria**** |
| --- | --- |
| Pre s-RARP PSA <10ng/ml | Pre s-RARP PSA <10ng/ml |
| Pathological ISUP score ≤2/3 at initial prostate cancer diagnosis | Pathological ISUP score ≤2/3 at initial prostate cancer diagnosis |
| cT1-2 stage at initial prostate cancer diagnosis | cT1-2 stage at initial prostate cancer diagnosis |
| cN0/ cM0 staging | cN0/ cM0 staging |
|  | ISUP score ≤3 prior to s-RARP |
|  | Time interval between initial treatment and s-RARP >12 months |
